# Early Gastric Cancer: Current Advances of Endoscopic Diagnosis and Treatment

**DOI:** 10.1155/2016/9638041

**Published:** 2016-01-17

**Authors:** Linlin Zhu, Jinyu Qin, Jin Wang, Tianjiao Guo, Zijing Wang, Jinlin Yang

**Affiliations:** ^1^Department of Gastroenterology, West China Hospital of Sichuan University, No. 37 Guo Xue Xiang, Chengdu 610041, China; ^2^Department of Gastroenterology, Kanazawa University Hospital, Kanazawa, Ishikawa 920-8641, Japan

## Abstract

Endoscopy is a major method for early gastric cancer screening because of its high detection rate, but its diagnostic accuracy depends heavily on the availability of endoscopic instruments. Many novel endoscopic techniques have been shown to increase the diagnostic yield of early gastric cancer. With the improved detection rate of EGC, the endoscopic treatment has become widespread due to advances in the instruments available and endoscopist's experience. The aim of this review is to summarize frequently-used endoscopic diagnosis and treatment in early gastric cancer (EGC).

## 1. Introduction

Gastric cancer is the fourth most common cancer worldwide and the second leading cause of cancer death [1]. With the raised public awareness on early diagnosis and treatment of cancer as well as the development of endoscopic imaging and image enhanced techniques, such as magnification narrow-band imaging, chromoendoscopy, and confocal laser endomicroscopy, the proportion of early gastric cancer (EGC) at diagnosis is increasing. Early detection is essential for treatment. It has been shown that the prognosis of EGC is excellent with a 5-year survival rate of over 90% [2–4].

According to the PARIS classification of superficial neoplastic lesions in the digestive tract [5], type 0 is divided into three categories corresponding to protruding lesions (0-I), nonprotruding and nonexcavated lesions (0-II), and excavated lesions (0-III). Type 0-I is subdivided into pedunculated (0-Ip) and sessile (0-Is) lesions. Type 0-II is divided into three subtypes, a, b, and c, corresponding to slightly elevated, flat, and depressed lesions. Type 0-III is all ulcer. Mixed patterns with elevation and depression also occur and are classified into two groups: in 0-IIc+0-IIc lesions, most of the surface is depressed; elevation is present in a segment of the lesion at the periphery; in 0-IIa+0-IIc lesions, there is a central depression in a globally elevated lesion. The combined patterns of excavation and depression are termed 0-III+IIc or 0-IIc+III, depending on the respective surface of the ulcer and of the depressed area.

The highest risk of invasion is seen in protruding (0-I) or depressed (0-IIc) lesions. The relation between the depth of invasion into the submucosa and lymph node metastases was analyzed in 1091 cases at the National Cancer Center Hospital in Tokyo [5]. When the invasion was less than the cut-off limit of 500 *μ*m, the proportion was only 6%; beyond this limit it increased to 21% [5].

Techniques of endoscopic treatment for early gastric cancer include endoscopic mucosal resection (EMR) and endoscopic submucosal dissection (ESD). The results of EMR in treating EGC are comparable to that of surgery in selected cases. ESD has been shown to increase* en bloc *resection of lesions regardless of their size, location, or presence of scarring [2, 6, 7]. As a class of minimally invasive endoscopic techniques, ESD is characterized by fewer traumas and complications and better therapeutic effects.

## 2. Endoscopic Diagnosis

China guidelines “China Consensus for diagnosis, treatment and endoscopic screening of early gastric cancer, 2014” recommended the high risk populations: (1) >40 years; (2)* Helicobacter pylori* infection; (3) previous precancerous diseases such as chronic atrophic gastritis, gastric polyps, gastric ulcer, and pernicious anemia; and (4) other high risk factors such as alcohol, smoking, high salt, preserved food [12].

White light endoscopy (WLE) can only detect obvious morphological changes of neoplastic lesions, such as changes in their color (redness), surface contour, or dynamic response to air/gas insufflations [8]. EGC can be divided into 3 types: elevated, superficial, and depressed. The superficial type is further subdivided into superficial elevated, superficial flat, and superficial depressed. It is difficult to find superficial flat lesions in the conventional WLE, which often cause misdiagnosis and missed diagnosis. The most common lesions of EGC were usually manifested by erythema and erosion.

Endoscopic ultrasonography (EUS) is used to check the exact structure of each layer of the gastric wall. It could be used to evaluate the infiltration depth of EGC or judge the lymph node metastasis, providing evidence for therapeutic choice. Fluorescence endoscopy can identify precancerous and some hidden lesions on the basis of fluorescence. However, the high demand of the equipment results in higher inspection cost, making its routine clinical use impossible or limited.

This review aims to summarize frequently used endoscopic diagnosis and treatment methods in EGC, such as magnifying endoscopy with narrow-band imaging (ME-NBI), confocal laser endomicroscopy (CLE), EMR, and ESD.

### 2.1. Magnifying Endoscopy with Narrow-Band Imaging

Magnifying endoscopy with narrow-band imaging (ME-NBI) is a recently developed technique, which is the combination of magnification endoscopy and narrow-band imaging. It is widely used in the detection of EGC based on the basic microanatomical findings of the microvascular (MV) pattern and microsurface (MS) structures of the superficial mucosa. The gastric mucosa is composed of glandular epithelium,which is different from the normal glands. There were many different classification systems to describe the correlation between the microanatomy and actual images visualized using ME-NBI.

The MV and MS patterns were reported by Yao and others [8, 13–15], where three microvascular/microsurface patterns were described: regular, irregular, and absent (Table 1). According to these MS/MV patterns, one could differentiate gastric low-grade adenoma from EGC or determine the lateral extent and the tumor invasion depth of early gastric cancer for curative endoscopic resection [11]. The criteria for diagnosing gastric cancer depend on the presence of irregular MS/MV pattern with a demarcation line [8]. It is worth mentioning that 97% of EGC fits the above criteria [16]. Kaise et al. examined the significance of various MS and MV changes, such as the disappearance of the MS pattern, a change in vessel caliber, and heterogeneity in appearance. These criteria determined the diagnosis of cancer with a sensitivity of 69.1% and a specificity of 85.3% [10]. To differentiate between adenocarcinoma and undifferentiated adenocarcinoma, Nakayoshi et al. classified the MV patterns of superficial gastric cancers into three groups: fine network pattern, corkscrew pattern, and unclassified pattern [11, 17]. Yokoyama et al. found that the intralobular loop (ILL) pattern and the presence of fine network or corkscrew vessels on MV pattern could be related to histological subtype, but it is not clear whether these criteria might be universally applied [9, 13].

Kobara et al. and Kikuchi et al. reported that ME-NBI can be used to determine the invasion depth in gastric cancer [7, 18]. Kobara et al. described three indicators: nonstructure, scattery, and multicaliber vessels. They suggested that the presence of all three indicators or the presence of two or more indicators with ME-NBI should be considered as diagnostic criteria of submucosa 2 (SM2) for depressed gastric cancer [18]. Kikuchi et al. found that the presence of dilated vessels (vessels with a diameter 3 times larger than that of the irregular microvessels that are frequently observed in the lesions) should be used to predict submucosal carcinoma; the specificity and negative predictive value (NPV) were close to 90%, but the sensitivity of dilated vessels was only 37.5% [7]. Kiyotoki et al. compared ME-NBI and indigo carmine chromoendoscopy (ICC) to evaluate the tumor margins and found that the accurate rate which making of ME-NBI was significantly higher than that of the ICC (97.4% versus 77.8%; *P* = 0.009) [19].

### 2.2. Confocal Laser Endomicroscopy

Confocal laser endomicroscopy (CLE) is a newly developed endoscopic imaging technology, which produces 1000-fold magnification cross-sectional images of the GI surface and subsurface tissue. It has the ability to provide a direct histological observation of the in vivo tissue without the need for biopsy and to differentiate malignant from benign lesions in real time at the cellular level [20]. Gastric pit is the smallest unit of the gastric mucosal surface and the smallest structural unit in the confocal images. It presents as different images in different disease states [21]. According to the classification of gastric pit patterns by Zhang et al. [20], the gastric pit patterns were divided into 7 types. Normal mucosa with fundic glands mainly contains round pits (type A). Type G is usually found on gastric cancer under CLE images, whose sensitivity and specificity for gastric cancer were 90% and 99.4%, respectively. Type G1 is defined as the loss of normal gastric pits accompanied by the appearance of diffusely atypical cells, such as signet ring cell cancer and low differentiated tubular adenocarcinoma. Type G2 is manifested by the loss of normal pits with the appearance of atypical glands, mainly in well differentiated tubular adenocarcinoma [20, 22].

CLE has great advantage on microvascular imaging, since blood vessels of normal and cancerous mucosa have different characteristics under CLE [23]. At present, the cellular changes and the tissue and vascular structure of CLE in the diagnosis of gastric mucosal lesions were based on sodium fluorescein staining. A prospective study involving 1786 patients was carried out by Li et al. to evaluate the validity and reliability of the CLE in the identification of gastric superficial cancerous lesions. They found that CLE diagnosis for early gastric cancers had high sensitivity (88.1%) and specificity (98.6%) [24]. Kakeji et al. examined normal and 27 gastric cancer tissues ex vivo. Compared to histology outcomes, CLE had a high diagnostic accuracy of 96.4%, and the sensitivity and specificity were 92.6% and 100%, respectively [25]. Kitabatake et al. obtained in vivo CLE images from normal mucosa and cancerous lesions in 27 patients with EGC and demonstrated sensitivity of 81.8%, specificity of 97.6%, and accuracy of 94.2% [26].

### 2.3. Endoscopic Treatment

The new NCCN guideline [27] recommended that EMR or ESD of early stage gastric cancer can be considered as adequate therapy when the lesion is ≤2 cm in diameter; it is shown on histopathology that it is well or moderately well differentiated, does not penetrate beyond the superficial submucosa, does not exhibit lymphovascular invasion, and has clear lateral and deep margins. But the guidelines did not specify when is EMR and when is ESD indicated.

The following are risk factors in case of endoscopic treatment: (1) failure of the lesion to lift after injection of saline into the submucosa (the nonlifting sign), (2) early gastric cancer with lymph node metastasis, (3) cancer invasion of the muscularis propria, and (4) severe coagulation dysfunction. Age is not a risk factor, except for severe organ failure. Anticoagulation should be stopped 5–7 days before the procedure [28].

### 2.4. Endoscopic Mucosal Resection (EMR)

Endoscopic mucosal resection was first introduced for endoscopic therapy in 1984 by using the strip biopsy method (two-channel method) [29, 30]. The operation process included submucosal injection under the lesion, snaring, and removing the lesion. This injection-snaring method is simple and convenient. However, it is difficult to trap flat type lesions. Furthermore, the steel wire slips easily, which may lead to incomplete resection and local recurrence [31]. EMR after circumferential precutting was described by Hirao et al. in 1988 [30, 32]. The injection-precutting-snaring (EMR-P) technique refers to submucosal injection of hypertonic saline mixed with diluted epinephrine, cut around the lesion with a needle knife, and removal of the lesion by a snare. In summary, the injection-snaring and injection-precutting-snaring techniques are noninhalation methods of EMR. The inhalation techniques of EMR include EMR with a cap (EMR-C) and EMR with ligation (EMR-L). EMR-C was developed in 1992 [33]. This technique can safely remove intramucosal cancers 2 cm or less in diameter by using transparent plastic cap that is connected to the tip of an endoscope. The procedure includes submucosal injection, suction into the cap, and snare and resection [2, 34]. The operation process of EMR with ligation (EMR-L) is similar to that of EMR-C. The equipment of EMR-L is a standard variceal ligation device. After sucking the lesion into the cap, lodged band is deployed underneath the lesion, and then the banded lesion is snared and removed [2, 35]. The traditional EMR methods (mentioned above) could not remove huge flat lesions for more than 2 cm one-time. This kind of lesions could be resection in several parts by endoscopy piecemeal mucosal resection (EPMR). However, it is difficult to splice the resected samples in vitro and evaluate the efficacy of radical resection after EPMR.

The Japan Gastroenterological Endoscopy Society (JGES) in collaboration with the Japanese Gastric Cancer Association (JGCA) has created guidelines for ESD and EMR for the treatment of EGC in 2015 [36]. The guidelines recommend that endoscopic resection should be carried out when the likelihood of lymph node metastasis is extremely low and lesion size and site are amenable to resection* en bloc*. Endoscopic therapy (EMR or ESD) is absolutely indicated in macroscopically intramucosal (cT1a) differentiated carcinomas measuring less than 2 cm in diameter. The macroscopic type does not matter, but there must be no finding of ulceration (scar), that is, UL(–).

The outcomes of EMR showed 56%–75.8%* en bloc* rates and 66.1%–77.6% complete resection rates in foreign countries [37–41]. A multicenter study on EMR in the treatment of early gastric cancer from Japan found that the complete resection rate is relevant to the lesion size; the complete resection rate of lesions less than 1 cm was 82.4%, while only 16.2% of those larger than 2 cm were resected completely [37]. Another Japanese literature reported that both 5-year and 10-year survival rates of patients with mucosal EGC less than 2 cm that were completely removed by EMR were 99% [2, 42]. The risk of local recurrence after EMR ranged from 2% to 35% in Japan [38]. Horiki et al. found that the local recurrence rate of early gastric cancer after piecemeal EMR was 30% (95% CI, 20–40%) at both 5 and 10 years [43]. Additional surgery was performed soon after the initial EMR if a resection margin was clearly positive for cancer. Furthermore, identification of the resection margin is a problem in EPMR.

### 2.5. Endoscopic Submucosal Dissection (ESD)

With the limitations of EMR resection of lesions, people seek to find new endoscopic techniques to remove larger tissues. Endoscopic submucosal dissection (ESD) permits* en bloc* resection of larger lesions [2, 44–50]. The steps of this endoscopic technique consist of marking, submucosal injection, circumferential mucosal precutting, dissection, and dealing with wound. The current indications for ESD are based on the criteria reported by Gotoda and colleagues, which include an ulcerative mucosal EGC < 3 cm in diameter or a submucosal invasion depth of the EGC ≤3 cm [51].

For ESD indications, according to the recent Japanese Gastric Cancer guidelines in 2015 [36] and ESMO-ESSO-ESTRO in 2013, in addition to EMR's indications, the ESD's indications include the following:

1. UL(–) cT1a differentiated carcinomas greater than 2 cm in diameter.
2. UL(+) cT1a differentiated carcinomas less than 3 cm in diameter.
3. UL(–) cT1a undifferentiated carcinomas less than 2 cm in diameter.
4. The extremely low risk of lymph node metastasis and the possibility of it becoming reasonable to expand the indications when vascular infiltration (ly, v) is absent together with the above-mentioned criteria.
5. The possibility of dealing with subsequent locally recurrent intramucosal cancers under expanded indications (evidence level V, grade of recommendation C1) if a lesion falls within the indication criteria at the initial ESD or EMR.

According to the literature, the* en bloc *resection rate of ESD for early gastric cancer was 94.9%–97.7% and 5-year survival rate was 83.1%–97.1% [52–57]. A multicenter retrospective study comparing EMR and ESD with resection in early gastric cancer reported that the one-piece resection rate with ESD was significantly higher than that with EMR (92.7% versus 56%) [41]. The incidence of perforation was 3.6% with ESD and 1.2% with EMR, although the complications were managed endoscopically. The 3-year cumulative residual-free/recurrence-free rate of ESD was 97.6%. In another study stated by Toyonaga et al. involving 1136 patients with gastric cancer, the* en bloc* resection was 97.1%, bleeding and perforation rates were 3.6% and 1.8%, and the 3-year and 5-year survival rates were 91.7% and 88.1% [58].

The mainly postoperative complications of ESD included bleeding, perforation, and stenosis. Acute intraoperative bleeding rate was 3.1–15.6% and the rate of delayed bleeding was 3.1% ~ 15.6% [59]. Bleeding may be associated with the size of lesions more than 4 cm or the thick submucosal blood vessels located in the upper two-thirds of the stomach [60, 61]. Perforation rate of ESD was 1.2%–4.1%; the risk factor of perforation is that the lesions more than 2 cm [59]. According to the study carried out by Coda et al., the stenosis rate after gastric body ESD was 17% (7/41), while 7% (8/115) pylorus stenosis occurred after ESD [62]. The study identified the following as the risk factors of stenosis, the surface of the mucosal circumferential defect that achieved to more than 3/4, or the length of the removal mucosal more than 5 cm.

As the kind of knives and the skill level of the operators were different, the proper number of cases required to gain adequate experience for ESD remains debatable [63–65]. Several studies suggested that experience comprising at least 30 cases overall and 30 cases in the lower third of the stomach [66] are needed for a beginner/trainee. Others suggested that a beginner could begin with lesions in the lower part of the stomach after 30 supervised ESD procedures [63]. A recent study suggested that experience with 30 procedures was not enough to complete all gastric ESDs without expert help for novice operators [67].

Procedure time was suggested as a marker of proficiency of the ESD. Bleeding control skills during submucosal dissection are a key feature of ESD [65, 67]. Hong et al. investigate the learning curve of ESD of gastric neoplasms by assessing the following parameters:* en bloc* resection rate, complete resection rate, duration and speed of procedure time, and related complications [68]. They found that the procedure time was significantly longer for lesions in the upper third of the stomach compared with lesions located in the middle third and lower third of the stomach (*P* = 0.01 and 0.01). Specimen size over 1501 mm^2^ was correlated with a longer procedure time in comparison with specimen size under 500, 501–1000, and 1001–1500 mm^2^ (*P* = 0.02, *P* < 0.01, and *P* < 0.01).* En bloc* resection rates and complete resection rates were not significantly related to the sequences of ESD procedures. The frequency of bleeding and perforation was not related to the sequence of treatment cases. This study suggested that novice operators begin with cases involving easy sites and small areas, which could lead to a higher complete resection rate compared with expert operators [68].

It is difficult to remove large lesions for ESD, as the lifting effect of the submucosal injection is less obvious after the circumferential incision than before it; the endoscopic view is obstructed or reduced when the resection reaches the central portion due to the confined space and contraction of the resected mucosa [69]. Local resection can be less accurate at evaluating the exact status of lymphovascular invasion and lymph node metastasis than at surgery [70]. It causes additional gastrectomy if the depth of invasion is deeper than the SM2 layer.

Endoscopic submucosal tunnel dissect (ESTD) ion was first introduced by Linghu et al. as a new strategy for rapid resection of large esophageal neoplasms [69]. A tunnel was established between the mucosa and the muscularis propria; then the lesions were resected rapidly. The use of ESTD to remove EGC has limited indications such as EGC with severe fibrosis due to previous ESD or severe ulceration and achievement of a sufficient resection margin because of submucosal invasion [71]. Choi et al. [71] reported two cases of ulcerative early gastric cancer with submucosal fibrosis that were treated by endoscopic submucosal tunnel dissection. It cannot be denied that ESTD has some advantages. For example, bleeding is easier to control due to the clearly exposed blood vessels, and the operation time is shortened. More research is required to assess the safety and effectiveness of the ESTD for resection of EGC.

## 3. Conclusion

In summary, with the development of endoscopic techniques, more prospective studies with high-quality designs should be performed to evaluate the diagnostic accuracy of these new endoscopic imaging techniques to add up the therapeutic outcomes, survival rates, and complication rates and to standardize the procedures and develop a learning system which is widely acceptable by endoscopists in the future.

## Figures and Tables

**Table 1:** Microvascular/microsurface patterns.

Study	Microvascular pattern	
Yao, 2013 [8]	Regular microvascular pattern	Closed- or open-looped with a uniform shape Homogeneous morphology Symmetrical distribution Regular arrangement
	Irregular microvascular pattern	Different shapes: closed-looped (polygonal), open-looped, tortuous, branched, bizarrely shaped Heterogeneous morphology Asymmetrical distribution Irregular arrangement
	Absent microvascular pattern	Subepithelial microvascular pattern is obscured Presence of a white opaque substance (WOS)

	Microsurface pattern	

Yao, 2013 [8]	Regular microsurface pattern	Shape of marginal crypt epithelium: uniform linear/curved/oval/circular structure Homogeneous morphology Symmetrical distribution Regular arrangement Regular WOS
	Irregular microsurface pattern	Shape of marginal crypt epithelium: irregular linear/curved/oval/circular structure Heterogeneous morphology Asymmetrical distribution Irregular arrangement Irregular WOS
	Absent microsurface pattern	No marginal crypt epithelial structure No WOS are visible

Yokoyama et al., 2010 [9]	Abnormal microvascular irregular superficial glandular pattern	Fine network pattern Corkscrew pattern Intralobular loop pattern-1 Intralobular loop pattern-2

	Microvascular features	Fine mucosal structural features

Kaise et al., 2009 [10]	Dilation Abrupt caliber alteration	Absence
	Denseness Heterogeneity in shape	Micrification
	Tortuousness	Heterogeneity

Nakayoshi et al., 2004 [11]	Microvascular pattern	A fine network pattern A corkscrew pattern An unclassified pattern
